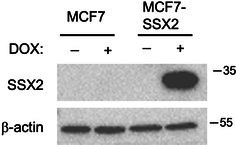# Correction to “Ectopic expression of cancer/testis antigen SSX2 induces DNA damage and promotes genomic instability”

**DOI:** 10.1002/1878-0261.70253

**Published:** 2026-04-27

Greve KB, Lindgreen JN, Terp MG, Pedersen CB, Schmidt S, Mollenhauer J, Kristensen SB, Andersen RS, Relster MM, Ditzel HJ, Gjerstorff MF. Ectopic expression of cancer/testis antigen SSX2 induces DNA damage and promotes genomic instability. *Mol Oncol.* 2015;**9**(2):437–49. https://doi.org/10.1016/j.molonc.2014.09.001

In this article, an error occurred during figure assembly that resulted in the Western blot images in Fig. 1E being published with an incorrect β‐actin control.

The authors have provided a technical replicate for the Western blot experiment along with a corrected version of Figure 1E, as shown below. The revised data are consistent with the findings reported in the original manuscript and confirm that SSX2 is not expressed in MCF7 cells but is induced in MCF7‐SSX2 cells.

The authors agree to this corrigendum and confirm that these changes do not affect the conclusions of the article. The authors apologize for any inconvenience caused.

Figure 1E: